# *Theobromacacao* Criollo var. Beans: Biological Properties and Chemical Profile

**DOI:** 10.3390/foods10030571

**Published:** 2021-03-09

**Authors:** Margherita Lavorgna, Severina Pacifico, Roberta Nugnes, Chiara Russo, Elena Orlo, Simona Piccolella, Marina Isidori

**Affiliations:** Department of Environmental, Biological and Pharmaceutical Sciences and Technologies, University of Campania “Luigi Vanvitelli”, Via Vivaldi 43, 81100 Caserta, Italy; margherita.lavorgna@unicampania.it (M.L.); severina.pacifico@unicampania.it (S.P.); roberta.nugnes@unicampania.it (R.N.); elena.orlo@unicampania.it (E.O.); simona.piccolella@unicampania.it (S.P.); marina.isidori@unicampania.it (M.I.)

**Keywords:** criollo, cocoa beans, DPPH/ABTS assay, antimutagenicity/antigenotoxicity, cancer cells, chemical characterization

## Abstract

*Theobroma cacao* provides precious products such as polyphenol-rich beans that are useful for nutraceutical purposes. The geographical area may influence the chemical composition of raw cocoa beans in terms of the polyphenols and biological qualities of the products. This work aimed to investigate the biological properties and the chemical composition of two different samples of Criollo var. cocoa raw beans coming from two areas (Indonesia; Peru). Beans underwent biphasic extraction obtaining lipophilic and hydroalcoholic extracts. The extracts were tested for antiradical, antimutagenic, and antigenotoxic effects. Cell viability inhibition toward breast, gastric/esophageal colorectal adenocarcinoma, and hepatoblastoma human cell lines was evaluated. Extracts were chemically investigated through UV-Vis spectroscopy and ultra-high-pressure liquid chromatography electrospray ionization quadrupole time-of-flight mass spectrometry (UHPLC-ESI-Q*q*TOF MS/MS). Results showed that the Indonesian bean hydroalcoholic extracts were able to scavenge 2′-azino-bis (3-ethylbenzothiazoline-6-sulfonic acid) diammonium salt (ABTS) cation radical better than the Peruvian hydroalcoholic extracts (ECs_50_: 72.63 vs. 322.20 μg/mL). Extracts showed antimutagenic and antigenotoxic activity. The viability inhibitory effect on breast and hepatic cancer cells was reached only for the Indonesian hydroalcoholic extracts at hundreds of μg/mL. Phenylpropenoyl-L-amino acids, hydroxycinnamoyl aminoacids conjugates, and procyanidin compounds were found mainly in the hydroalcoholic extracts, whereas fatty acids and lyso-phospholipids were found mainly in lipophilic fractions. Fatty acid and (*epi*)catechins appeared to be affected by different environmental conditions of the geographical areas.

## 1. Introduction

A diet rich in plant-derived products positively affects human life ensuring the constant intake and supplementation of beneficial phytochemicals. These compounds are claimed to maintain health and prevent illness, and they are hypothesized as a promising strategy in the management of several chronic degenerative diseases. Among these, in recent years, cancer is becoming an evident issue. According to the World Health Organization (WHO, 2018), cancer is the second chronic disease (9.6 million deaths), and cancer of breast (627,000 deaths), liver (782,000 deaths), stomach (783,000 deaths), and of colorectal (862,000 deaths) are among the most common causes of cancer death. Malignant transformation and uncontrolled growth of cells may be caused by the production of free radicals, mutations, and altered gene expression. Hence, antioxidant, antimutagenic, and antigenotoxic substances may play a major role in the primary prevention of cancer development [1,2]. Among phytochemicals, polyphenols are receiving increasing attention due to their preventive efficacy in offsetting oxidant species over-genesis in normal cells, and with the potential ability to arrest or reverse oxidative stress-related diseases [3]. Thus, polyphenol-rich plant products, in their crude or processed form, need to be thoroughly investigated also considering the great variability of these compounds in response to various factors. In fact, several biotic and abiotic factors influence polyphenol content, and extractive processing massively impacts the formulation of polyphenol-enriched extracts that are useful for nutraceutical purposes. In this context, taking into account the imperishable cocoa consumption, it is reasonable to assume that cocoa, because of its polyphenolic compounds, may contribute to the prevention or treatment of cancer diseases [4,5].

Basically, the tropical cacao plant provides precious edible products: cacao beans, which can be harvested and consumed directly, or roasted and powdered as cocoa. This latter can be processed as chocolate, which was considered the “drink of the Gods”, so much so that the scientific plant name, *Theobroma cacao*, was taken from the Greek words: *theo* (God) and *broma* (drink) [6]. In addition to cocoa’s health benefits, its flavor is one of the most significant products because of different characteristics such as processing steps (fermentation, drying, and roasting), and the origin of the cocoa plant geographical area [7], which influence the starting chemical composition of raw cocoa beans in terms of polyphenols, compromising the antioxidant activity and the quality of the final products [8].

Among cocoa pure lines, Criollo is one of the varieties of high quality [9]. Thus, the aim of this work was to investigate the biological properties and the chemical composition of two different samples of Criollo var. cocoa raw beans coming from two different geographical areas (Indonesia, Asia; Peru, South America). The samples were tested for their radical scavenging power against 2,2-diphenyl-1-picrylhydrazyl (DPPH) and 2′-azino-bis (3-ethylbenzothiazoline-6-sulfonic acid) diammonium salt (ABTS) radical, for their antimutagenic effect in *S. typhimurium* TA98 and TA100 strains, auxotroph for histidine (His^−^) by the *Salmonella* mutagenicity assay (Ames test), and for their antigenotoxic effect in *S. typhimurium* TA1535/pSK1002 strain (Umu test). Finally, the cell viability inhibition effect toward breast adenocarcinoma (MCF-7), gastric–esophageal adenocarcinoma (OE19), hepatoblastoma (Hep-G2), and colorectal adenocarcinoma (Caco-2) human cell lines (four among the most widespread cancers) was assessed by 3-(4,5-dimethylthiazol-2-yl)-2,5diphenyl-tetrazolium bromide (MTT) assay. Moreover, in order to study the chemical basis of the results obtained, the chemical composition of cocoa beans was investigated through ultra-high-pressure liquid chromatography electrospray ionization quadrupole time-of-flight mass spectrometry (UHPLC-ESI-Q*q*TOF MS/MS).

## 2. Materials and Methods

### 2.1. Reagents

Dimethylsulfoxide (DMSO), 2,2-diphenyl-1-picrylhydrazyl (DPPH, CAS: 1898-66-4), 2′-azino-bis (3-ethylbenzothiazoline-6-sulfonic acid) diammonium salt (ABTS, CAS: 30931-67-0), potassium persulfate (CAS: 7727-21-1), 6-hydroxy-2,5,7,8-tetramethylchroman-2-carboxylic acid (Trolox, CAS: 53188-07-1), 3-(4,5-dimethylthiazol-2-yl)-2,5diphenyl-tetrazolium bromide (MTT, CAS: 289-93-1), 4-nitroquinoline-1-oxide (4-NQO, CAS: 56-57-5), L-histidine (CAS: 71-00-1), biotin (CAS: 58-85-5), *O*-nitrophenyl-β-D-galactopyranoside (ONPG, CAS: 369-07-3), 2-nitrofluoren (2-NF, CAS: 607-57-8) were supplied by from Sigma-Aldrich (Milano, Italy). Sodium azide (SOD, CAS: 26628-22-8) was from JT Baker (Milano, Italy). Dulbecco’s Modified Eagle’s Medium phenol red-free (DMEM), *N*-(2-hydroxyethyl)piperazine-*N′*-(2-ethanesulfonic acid) (HEPES), fetal bovine serum (FBS), penicillin/streptomycin (10,000 U/mL), L-glutamine, Roswell Park Memorial Institute Medium (RPMI 1640), non-essential amino acid (MEM, 100×), Dulbecco’s phosphate-buffered saline (DPBS), trypsin-EDTA (10×) were purchased by Lonza Bio Whittaker (Verviers, Belgium).

### 2.2. Sample Collection and Extraction Procedure

Beans of *Theobroma cacao* were purchased from NaturaSì, Italy. Two different samples of cocoa beans belonging to the same variety (Criollo var.) and coming from two different geographical areas were chosen: Asia and South America. Thus, the two cocoa beans samples were identified as follows: Indonesian cocoa beans (ICB) and Peruvian cocoa beans (PCB). Beans were pulverized through a knife mill and stored at 4 °C until extraction. Biphasic extraction, which favorably allows water-soluble compounds to be separated from apolar ones using two immiscible solvents, was carried out. To this purpose, cocoa beans (15 g each) were poured into a methanol/chloroform/water (2:1:1, *v*:*v*:*v*) solution with a 1:4 ratio (1 g per 4 mL of extracting solution), and the partition of a chloroform (O) layer, and an hydroalcoholic (hA) fraction was achieved. The fractions were separately collected and dried using an IKA rotary evaporator at 40 °C. For Indonesian cocoa beans, ICB_O_ (1.59 g) and ICB_hA_ (0.40 g) fractions were obtained, whereas for Peruvian matrix, the fractions PCB_O_ and PCB_hA_ were 1.47 and 0.39 g, respectively. Stock solutions for biological evaluations were prepared dissolving obtained chloroform and hydroalcoholic fractions in DMSO and methanol respectively, and solvents percentage in test solutions were <0.1%.

### 2.3. Antiradical Activity

#### 2.3.1. ABTS Assay

ABTS assay was performed following the protocol described by Re et al. [10], with some modifications [11]. The ABTS**^·^**^+^ was generated by adding the potassium persulphate (140 mM K_2_S_2_O_8_) to the ABTS solution (7 mM) for 12–16 h in the dark. Then, the solution was diluted using PBS to reach the absorbance of 0.7 ± 0.02 OD at 734 nm. Therefore, 100 μL of cocoa beans extracts (10, 25, 50, 75, 100, 200, 500 μg/mL—chosen after range-finding tests) were added to 1 mL of ABTS solution, and the absorbance was measured after 6 min in reference to a negative control (1 mL of ABTS^+^ solution and 100 μL of distilled water). Distilled water was used for the blank and Trolox^®^ was used as standard antioxidant. In addition, solvent controls were prepared. The ABTS^+^ scavenging percentage (RS%) was calculated in line with Rakholiya and colleagues [12]:

RS (%) = [(OD cation radical − OD sample)/(OD cation radical)] × 100.(1)

Results, coming from three independent experiments, were expressed as the concentration of samples to scavenge the ABTS^+^ of 50% (EC_50_) and as Trolox Equivalent Antioxidant Capacity (TEAC) values calculated according to Shimamura and colleagues [13]:

TEAC = EC_50_ Trolox/EC_50_ sample. (2)

#### 2.3.2. DPPH Assay

DPPH radical scavenging activity was performed following the protocol described by Brand-Williams et al. [14] and Pacifico et al. [15]. First, 60 μL of cocoa beans extracts (10, 25, 50, 75, 100, 200, 500 μg/mL) was dissolved in 940 μL of DPPH· methanol solution (101.43 μM) at room temperature. Negative control and blank were prepared by adding 60 μL of distilled water to 940 μL of DPPH solution or of methanol, respectively. The absorbance was measured at 515 nm after 30 min and RS% and TEAC values were calculated as described above.

### 2.4. Antimutagenicity and Antigenotoxicity Activity

Before beginning antimutagenic and antigenotoxic assays, Ames and Umu tests were performed also to assess the possible mutagenicity and genotoxicity of cocoa beans extracts.

#### 2.4.1. Salmonella Mutagenicity Assay (Ames Test)

An Ames test was performed to test the antimutagenicity of cocoa beans extracts. Two Salmonella strains were used: the TA98 strain to remark frame-shift mutations and TA100 to evaluate base-pair substitutions due to missense mutations [16,17]. Both strains were from the permanent collection of the Laboratory of Hygiene and Environmental Toxicology, University of Campania ‘Luigi Vanvitelli’, Italy.

For the mutagenicity test, 100 μL of extracts (1000 μg/mL, chosen as the highest concentration), 100 μL of *S*. *typhimurium* TA98 or TA100 cultured overnight (10^8^ cells), and 500 μL of 0.1 M phosphate buffer (pH 7.4) were added to 2.5 mL of 0.5 M histidine–biotin top agar and then poured into minimal glucose agar in triplicate and incubated for 72 h at 37 °C. Furthermore, saline solution (0.9% NaCl) was used as negative control while 2-nitrofluoren (2-NF) at 2.5, 5, and 10 μg/mL and sodium azide (SOD) at 5, 10, and 20 μg/mL were used as standard mutagens for TA98 and TA100, respectively. Solvent controls were prepared also. After incubation, induced His^+^ revertants were counted after 72 h instead of 48 h to simplify the reading. The mutagenic ratio (MR) was calculated as follows:

MR = mean number revertans of sample/mean number revertans of negative control(3)

A sample was considered mutagenic when MR ≥ 2.

Differently, for the antimutagenicity assessment, the samples (10, 50, and 100 μg/mL, with the highest concentration equal to 1/10 of the concentration used to evaluate the possible mutagenic effect, 1000 μg/mL) were previously co-incubated with increasing concentrations of standard mutagens (2.5, 10 μg/mL of 2-NF for TA98 and 5, 20 μg/mL of SOD for TA100) for 2 h at 37 °C. The results, coming from three independent experiments, were expressed as the percentage of the ability of the extracts to inhibit the action of the mutagen, which was calculated as suggested by Resende and colleagues [18]:

Inhibition (%) = 100 − [(T/M) × 100](4)

where T is the mean number of revertant colonies in the plates containing both mutagen and test extracts, and M is the mean number of revertant colonies in the plates containing only the mutagen.

According to Resende and colleagues [18], an inhibition lower than 25% was considered to indicate no antimutagenic effect, an inhibition value between 25% and 40% was considered to indicate a moderate effect, and values greater than 40% were considered to indicate strong antimutagenicity.

#### 2.4.2. Salmonella Genotoxicity Assay (Umu Test)

An umu bioassay [19] was performed in *S. typhimurium* TA1535/pSK1002 strain to evaluate the genotoxicity of the samples by observing the induction of the SOS-repair system. The plasmid pSK1002 carries the *umuC* gene fused with the *LacZ*, which is a structural gene for β-galactosidase. Its activity is strictly dependent on *umuC* expression in response to specific DNA-damaging agents.

The *S. typhimurium* strain (optical density ≥ 800 Formazine Nephelometric Units, FNU) was purchased by EBPI (Burlington, Ontario, Canada). Before performing antigenoxicity assays, the samples were tested to exclude their possible genotoxic effect. Thus, 180 μL of the cocoa beans’ extracts (1000 μg/mL, chosen as the highest concentration) and 20 μL TGA (tryptone, glucose, and ampicillin) medium (ten-fold concentrated) were mixed with 70 μL of exponentially growing bacteria (340–350 FNU) into each well of the 96-well microplate (plate A), in triplicates. In addition, solvent controls were prepared. Moreover, saline solution (0.9% NaCl) and 4-nitroquinoline (4-NQO, 0.05 μg/mL) were used as negative and positive controls, respectively. The blank was prepared using 70 μL of TGA medium instead of the 70 μL of the bacterial culture. The plate was incubated for 2 h at 37 °C in shaking condition at 150 rpm, and then, 30 μL from each well were transferred in a new microplate (plate B) containing fresh medium and re-incubated for a second time. The absorbance at 620 nm was measured using the automated microplate reader (Synergy H1, Biotek, Winooski, VT, USA) to evaluate the density of the strain. Then, a new microplate (plate C) was prepared by mixing 30 μL from each well of plate B and 120 μL of B-buffer (0.1 M sodium phosphate pH 7.4, 10 mM KCl, 1 mM MgSO_4_, 1 mg/L β-mercaptoethanol, 10 μL of SDS 1 mg/mL). For the determination of β-galactosidase activity, the enzymatic reaction was activated by adding 30 μL of 4.5 mg/mL ONPG. After 30 min of incubation at 28 °C, 120 μL Na_2_CO_3_ (1 M) were added to stop the reaction. The absorbance was recorded at 420 nm. The β-galactosidase activity (βgal.unit) was calculated as follows:

βgal.unit = (A_420T_ − A_420B_)/(A_620T_ − A_620B_) (5)

where A_420_ is the absorbance at 420 nm relative to the enzymatic reaction intensity of samples (T) and blank (B), while A_620_ is the absorbance at 620 nm of bacteria growth of samples and blank.

An induction ratio (IR) was used to quantify the genotoxicity, and it was calculated as follows:

IR = (βgal.units_T_)/( βgal.units_N_) (6)

where T is absorbance at 420 nm of the sample and N is the absorbance of the negative control corrected for growth rate at 620 nm. When the IR value is equal to or higher than 1.5, the sample was considered genotoxic.

The antigenotoxicity test was assessed following the same procedure used for the genotoxicity. Three different concentrations of extracts (25, 50, and 100 μg/mL, with the highest concentration equal to 1/10 of the concentration used to evaluate the possible genotoxic effect, 1000 μg/mL) were pre-incubated for 2 h at 37 °C with 4-NQO, a standard genotoxin. The percentage of antigenotoxicity was calculated [20]:

Antigenotoxicity (%) = [1 − (βgal. unit_genotoxin+sample_/βgal. unit_genotoxin_)]∙100. (7)

Results came from three independent experiments, and extracts were considered as a neutral antigenotoxic when lower than 40%, medium between 40% and 70%, and strong above 70% [21].

### 2.5. Cell Viability Inhibition

#### 2.5.1. Cultivation of Human Cancer Cell Lines

Breast adenocarcinoma (MCF-7) as well as hepatoblastoma (Hep-G2) cell lines and gastric–esophageal adenocarcinoma (OE19) cell lines were respectively provided by Prof. Abbondanza and Prof. Morgillo (Department of Precision Medicine, University of Campania “Luigi Vanvitelli”, Italy). Colorectal adenocarcinoma (Caco-2) cell lines were provided by Prof. Potenza (Department of Environmental, Biological and Pharmaceutical Sciences and Technologies, University of Campania). Authentication, characterization, and mycoplasma testing were performed to check periodically the quality of cells.

MCF-7, Hep-G2, and Caco-2 cell lines were grown in RPMI containing 10% FBS, 2% HEPES, 2% L-glutamine, 1% penicillin–streptomycin, and only for Caco-2 was used 1% MEM, while the OE19 cell line was grown in DMEM high glucose under the same conditions. All cell lines were cultured in tissue culture flasks T-75 (Sarstedt, Verona, Italy) at 37 °C in a humidified atmosphere of 95% air plus 5% CO_2_. When cells reached 80–90% of confluence, they were washed with DPBS, detached with trypsin-EDTA, and centrifuged at 200× *g* for 5 min. In order to subculture the cells, they were counted under an optical microscope after using trypan blue vital dye.

#### 2.5.2. MTT Assay

The inhibition of viability of cancer cells, caused by cocoa beans extracts, was evaluated by MTT assay according to Baharum and colleagues [22] with slight modifications suggested by Lavorgna and co-authors [11]. This test was a sensible method to assess the cell viability for determining the activity of mitochondrial dehydrogenases to reduce MTT salts into formazan salts, obtaining a purple solution in living cells.

MCF-7, OE19, Hep-G2, and Caco-2 cell lines were plated (100 μL/well) in quadruplicate in 96-well plates at a density of 10^4^ cells/well and, after 24 h of incubation, the medium was removed from all wells, and 200 μL/well of cocoa beans extracts (0.9–1000 μg/mL, geometric progression of 2—chosen after range finding tests) or culture medium (negative control wells) was added. Solvent controls were prepared also. Plates were incubated for 72 h; then, 20 μL of MTT (5 mg/mL) was added to each well. After 4 h, the solution was removed, and 200 μL/well of isopropyl alcohol was used to dissolve the formazan salts. The absorbance was recorded at 590 nm using the automated microplate reader. Three independent assays were performed and the evaluation of the cell inhibitory was expressed as:

IC% = 1 − (extract absorbance/control absorbance) × 100.(8)

The concentration inhibiting the 50% cell growth rate (IC_50_) was calculated.

### 2.6. UV-Vis Spectroscopy for ICB and PCB Extracts’ Analysis

The UV-Vis spectra of extracts from both Indonesian and Peruvian cocoa beans were recorded using a Cary 100 UV-Visible Spectrophotometer by Agilent Technologies (Cernusco sul Naviglio, Milan, Italy) from 200 to 800 nm. Quartz cells (1 cm) were used for all absorbance measurements. The spectra were recorded in triplicate from 200 to 800 nm.

### 2.7. UHPLC-ESI-QqTOF-MS/MS Analysis

A Shimadzu NEXERA UHPLC system was used with a Luna^®^ Omega Polar C18 column (150 × 2.1 mm i.d., 1.6 μm particle size; Phenomenex, Torrance, CA, USA). The mobile phase consisted of a binary solution composed by water (solvent A) and acetonitrile (solvent B), which were both acidified with formic acid (0.1% *v*/*v*). For achieving hydroalcoholic extracts separation, gradient conditions were as follows: 0–5 min, linear from 5 to 15% B; 5–15 min, linear from 15 to 45% B; 15–16 min, from 45 to 75% B; 16–18.5 min, from 45 to 75% B; 18.5–19.5, isocratic 95% B. Then, the system was allowed to re-equilibrate 1.5 min before the next analysis. When organic fractions were investigated, gradient elution was as follows: 0–5 min, linear from 5 to 55% B; 5–10 min, linear from 55 to 75% B; 10–11 min, from 75 to 95% B; 11–12 min, isocratic 95% B. Then, at 12.01 min, the starting conditions were restored, and the column was allowed to re-equilibrate for 2 min. The total run time was 14 min. In both the cases, the injection volume was 2.0 μL, and the flow was set at 0.4 mL/min.

The AB SCIEX TripleTOF^®^ 4600 (AB Sciex, Concord, ON, Canada) system was combined with the UHPLC. It was equipped with a DuoSpray ion source, with the ESI probe used for MS investigations in both negative and positive ionization mode, and the APCI probe used for fully automatic mass calibration, using the Calibrant Delivery System (CDS). CDS injects a calibration solution matching the polarity of ionization and calibrates the mass axis of the analyzer in all scan functions (MS or MS/MS). For hydroalcoholic extracts analysis, data were collected by information-dependent acquisition (IDA) using a TOF-MS survey scan of 150–1750 Da (250 ms accumulation time) and eight dependent TOF-MS/MS scans of 80–1500 Da (100 ms accumulation time), using a collision energy (CE) of 45 V with a collision energy spread (CES) of 15 V. The other parameters were set as follows: declustering potential (DP), 70 V; ion spray voltage, −4500 V; ion spray heater, 600 °C; curtain gas, 35 psi; ion source gas, 60 psi. The full-scan TOF survey applied for apolar extracts was in the range 100–2000 Da, with eight IDA MS/MS scans. The declustering potential (DP) was set at 75 V. Data processing was performed using the PeakView^®^-Analyst^®^ TF 1.7 software.

### 2.8. Statistical Analysis

IC_50_ or EC_50_ values from biological assays were obtained by non-linear regression (log agonist vs. normalized response-variable slope) with a 95% confidence range using GraphPad Prism 5 analysis (Inc., San Diego, CA, USA). Furthermore, one-way ANOVA Dunnett’s comparison was used to evaluate the Lowest Observed Adverse Effect Concentrations (LOAECs) and statistical differences from controls were considered significant when *p* values were * *p* < 0.05, ** *p* < 0.001, and *** *p* < 0.0001.

## 3. Results

### 3.1. Antiradical Activity

EC_50_ and TEAC values of hydroalcoholic extracts of ICB and PCB samples, in DPPH and ABTS assays, were reported in Table 1. ICB_hA_ and PCB_hA_ samples were able to scavenge the two free radicals; nevertheless, the ICB_hA_ extract was more active than PCB_hA_ with EC_50_ values equal to 186 and 72.63 μg/mL, respectively obtained in DPPH and ABTS assays. Between the two antiradical assays, the DPPH assay was the most suitable as highlighted by TEAC values equal to 0.40 and 0.26 for ICB and PCB samples, respectively. Otherwise, organic fractions (ICB_O_ and PCB_O_) results were not shown because no effects up to the highest tested concentration (500 μg/mL) were obtained. No statistically significant differences were found comparing results coming from solvent control and negative control.

### 3.2. Mutagenicity/Genotoxicity

Ames and Umu tests were carried out to exclude the possible mutagenic and genotoxic effects of the ICB and PCB samples. Thus, they were tested at 1000 μg/mL, and results are reported in Table S1.

No mutagenic effect (MR < 2) in the *S. typhimurium* TA 98 and TA 100 strains and no genotoxic effect (IR < 1.5) in the *S. typhimurium* TA1535/pSK1002 strain were obtained.

### 3.3. Antimutagenicity

Antimutagenicity results were expressed as the mean of revertants/plates ± SD (*n* = 3) and as the inhibition rate percentage (% mean ± SD) (Table 2). When ICB and PCB samples (10, 50, 100 μg/mL) were co-incubated with 2-NF (2.5, 10 μg/mL) and SOD (5, 20 μg/mL), they showed statistically significant moderate and strong antimutagenic effects. Specifically, in TA98 *Salmonella* strain, when all concentrations of cocoa beans samples were co-incubated with 2-NF at 2.5 μg/mL, a strong antimutagenic activity (>40%) was observed. Otherwise, when cocoa samples were co-treated with the highest concentration of the standard mutagen (2-NF at 10 μg/mL), a strong antimutagenic activity occurred at their increasing concentrations. In the TA100 *Salmonella* strain, all concentrations of ICB and PCB induced a strong antimutagenic effect when co-treated with both concentrations of SOD. No statistically significant differences were found comparing results coming from solvent control and negative control.

### 3.4. Antigenotoxicity

In Table 3, the results of the antigenotoxic activity (coming from three independent experiments) were expressed as the mean of induction ratio (IR) ± SD and as antigenotoxicity (% mean ± SD). Significant differences (* *p*< 0.05, ** *p*< 0.001) were observed between IR values obtained from the single 4-NQO (0.05 μg/mL) and IR values obtained from ICB and PCB samples (100 μg/mL) co-treated with the standard genotoxin. Moreover, at the highest tested concentration, samples showed a moderate antigenotoxic activity inhibiting the 4-NQO-SOS response in *S.* *typhimurium* TA1535/pSK1002 from 40 to 70%. No statistically significant differences were found comparing results coming from solvent control and negative control.

### 3.5. Cell Viability

The results regarding the median viability inhibition and concentration–effect curves of MCF-7, OE19, Hep-G2, and Caco-2 cells exposed to ICB and PCB were reported in Table 4 and in Figure S1, respectively. Hydroalcoholic and organic fractions of both bean samples were active on the Caco-2 cell line with IC_50_ values that ranged from 104.90 to 234.00 μg/mL and with LOAEC values in the order of units of μg/mL for hydroalcoholic extracts and of dozens of μg/mL for organic extracts. Otherwise, for MCF-7 and Hep-G2, a median inhibitory effect was reached only in hydroalcoholic extracts (IC_50_ in the order of hundreds μg/mL) with a higher viability inhibition when exposed to ICB. However, a higher sensitivity of breast cancer cells was observed at the lowest adverse effect concentrations equal to 3.9 μg/mL for all extracts of both samples, differently from liver cancer cells whose lower sensitivity was generally observed at lower tested concentrations with higher LOAECs (31.2 μg/mL). OE19 cells were far less sensitive than all other cell lines when treated with both extracts of cocoa samples. No statistically significant differences were found comparing results coming from solvent control and negative control.

### 3.6. Compounds in Cocoa Beans Extracts from UV-Vis and UHPLC-ESI-QqTOF-MS/MS Analysis

**Figure 1:**
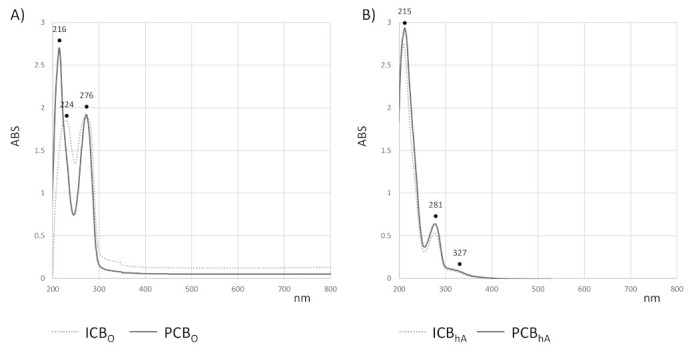
UV-Vis spectra. UV-Vis spectra of chloroform (**A**) and hydroalcoholic (**B**) extracts from Indonesian (dashed line) and Peruvian (continuous line) cocoa beans. Spectra are recorded in the range 200–800 nm. The bands’ wavelength is indicated.

In order to unravel the chemical composition of the cocoa beans extracts, the UV-Vis spectroscopic tool, together with UHPLC-ESI-Q*q*TOF-MS/MS analyses, was carried out. The UV-Vis absorption profile of each chloroform extract showed two bands (Figure 1, panel A). These latter bands were not fully overlapping. In fact, beyond the common absorption at 276 nm, the ICB_O_ extract is characterized by a band at 224 nm, which is not detectable from Peruvian cocoa beans lipophilic extract. Three bands at 215, 281, and 327 nm were observed for both the hydroalcoholic extracts (Figure 1, panel B), which are suggested to have a comparable chemical composition, which is mainly constituted by phenolic and flavonoid compounds. In fact, the absorptions detected are in line with the main absorption maxima of flavonoids, which are in the ranges 240–285 nm (A-ring benzoyl system, band II) and 300–400 nm (B-ring cinnamoyl system, band I).

UHPLC-ESI-Q*q*TOF-MS/MS analyses allowed us to better deepen into the chemical composition of all the prepared extracts, establishing their relative qualitative–quantitative differences.

#### 3.6.1. UHPLC-ESI-QqTOF-MS/MS Analyses of Hydroalcoholic Extracts

ICB_hA_ and PCB_hA_ fractions appeared to be constituted by thirty-two constituents. TOF-MS and TOF-MS/MS data, acquired in ESI negative ion mode, were listed in Table 5, together with molecular formulas, unsaturation degree (RDB—Ring and Double Bond) values, and mass accuracy. Compounds **1** and **2** are saccharides. In particular, the [M-H]^−^ ion of compound **1** at *m*/*z* 181.0716 was in accordance with the C_6_H_14_O_6_ molecular formula. This is likely due to a 6-member sugar alcohol as mannitol [23].

**Figure 2:**
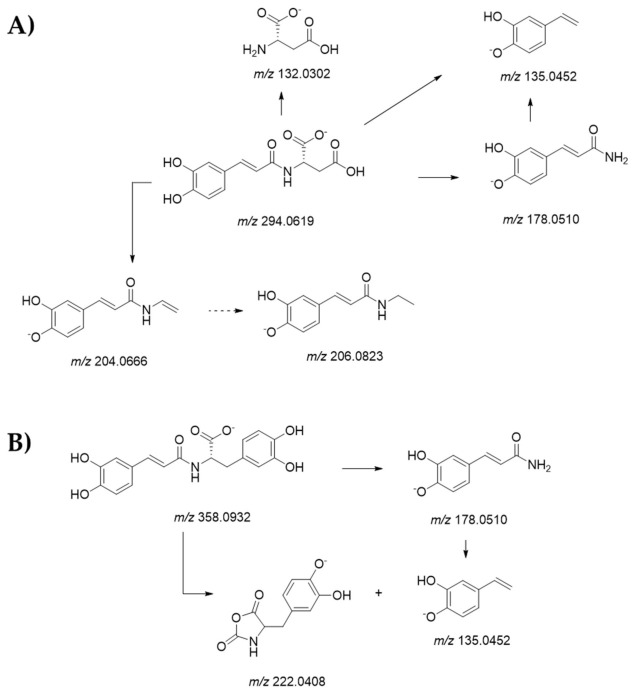
Fragmentation scheme of compounds. Proposed fragmentation scheme of compounds (**A**) **4** and (**B**) **10**. Theoretical *m*/*z* values are reported below each hypothesized structure.

Compound **2** was putatively identified as a disaccharide. In fact, its deprotonated molecular ion was at *m*/*z* 341.1094, according to C_12_H_22_O_11_ formula, and MS^2^ fragment ions at *m*/*z* 179.0553, 161.0459, and 113.0252 provided information about the sugar monomer identities. Compound **3** could be isocitric acid, as its [M-H]^−^ ion at *m*/*z* 191.0198 underwent dehydration, decarboxylation, and further dehydration to achieve the fragment ion at *m*/*z* 111.0087 [24]. Compounds **4**, **7**, **10**, **14**, and **22** were *N*-phenylpropenoyl-L-amino acids hydroxycinnamoyl amino acids conjugates. The TOF-MS experiment of compound **4** showed the [M-H]^−^ ion at *m*/*z* 294.0618, whereas the [2M-H]^−^ ion was also detectable at *m*/*z* 589.1321. The [M-H]^−^ ion underwent a double decarboxylation providing the TOF-MS^2^ fragment ions at *m*/*z* 206.0828 and 204.0697, or following N-CO α-cleavage gave the ion at *m*/*z* 132.0318 (base peak), likely corresponding to aspartate. The presence of this latter was further confirmed by the ion at *m*/*z* 115.0048, which is due to carboxyacrylate. As a result of the N-Cα bond breakdown, the fragment ion at *m*/*z* 178.0527 was due to the deprotonated amide of caffeic acid. The identity of the hydroxycinnamoyl moiety was also confirmed by the ions at *m*/*z* 161.0236 and 135.0466 (Figure 2A). A similar fragmentation pattern occurred for compound **7**, which was different from the previous one by phenolic residue, which was tentatively identified as coumaroyl. In fact, the [M-H]^−^ ion at *m*/*z* 278.0671 provided in the MS/MS experiment fragment ions at *m*/*z* 190.0857 (due to double decarboxylation) and 162.0560, which likely consisted in the deprotonated coumaramide. The detection of the ion at *m*/*z* 115.0032 suggested that aspartic acid was also in this case the amino acid residue.

Compound **10** was putatively identified as *N*-caffeoyl 3-hydroxytyrosine (clovamide), which was previously reported as a constituent in cocoa beans and hulls [25]. According to Bouchez et al. [26], the deprotonated molecular ion at *m*/*z* 358.0929, through CO-Cα’ bond cleavage, gave the ions at *m*/*z* 222.0396, which could correspond to the 4-((2,5-dioxooxazolidin-4-yl)methyl)-2-hydroxyphenolate and 135.0448 (base peak). This latter, together with the fragment ions at *m*/*z* 178.0503, and 161.0237, as well as those at *m*/*z* 134.6163, and 133.0301 confirmed the caffeoyl identity of the hydroxycinnamoyl moiety (Figure 2B). Finally, compounds **14** and **22** were tentatively identified as *N*-caffeoyl tyrosine and *N*-coumaroyl tyrosine, respectively. In the MS/MS spectrum of both compounds, the fragment ion at *m*/*z* 180.0675 (64) was identifying deprotonated tyrosine, whereas the base peak corresponds to the hydroxycinnamoyl moiety. These compounds are commonly known for their beneficial properties.

*N*-phenylpropenoyl amino acids, which are reported as the key contributors to the astringent taste of cocoa beans, were 1.33-fold higher in ICB_hA_ than PCB_hA_. Indeed, as it was expected, the great partest of the compounds belongs to the flavan-3-ol class, which constituted PCB_hA_ (77.3%) and ICB_hA_ (62.7%). Compound **5** was tentatively identified as gallocatechin, based on the comparison of its MS/MS data with those of an authentic standard compound, whereas compounds **8** and **9**, which showed the deprotonated ions at *m*/*z* 289.0720 and 289.0714, respectively, were identified as catechin and epicatechin. TOF-MS/MS spectra clearly displayed typical catechins’ neutral losses attributable to decarboxylation and A-ring cleavage, as well as the characteristic HRF ion at *m*/*z* 125.0247 and 125.0252. In the MS^2^ spectrum of compound **9**, the base peak at *m*/*z* 109.0308 could be due to deprotonated catechol moiety (B ring), whereas the ion at *m*/*z* 123.0461 was from the benzofurane-forming fission reaction [27].

Compounds **6**, **11**, **15**, and **29** are B-type procyanidins. In fact, the TOF-MS/MS of these dimers provided similar fragment ions, among which those at *m*/*z* 451, 425, 407, and 289 appeared as the most abundant. RDA fragmentation gave information about the hydroxylation of ring B. In fact, the MS^2^ ion at *m*/*z* 425 was produced by the loss of an RDA fragment, and the further water loss produced an ion at *m*/*z* 407. Furthermore, the fragment ion at *m*/*z* 289 corresponded to a quinone methide fragment, and it was in accordance with (*epi*)catechin as both top and base units. The heterocyclic ring fission mechanism promoted the catechol loss from the A-ring for providing the fragment ion at *m*/*z* 451 [3]. The TOF-MS and TOF-MS/MS spectra of compounds **23** and **31** were in accordance with an (*epi*)catechin ethyl dimer. In fact, the deprotonated molecular ion at *m*/*z* 605.1685 (**23**) and 605.1673 (**31**) underwent the typical 152 Da neutral loss for providing the ions at *m*/*z* 453.1197 and 453.1168, respectively. The QM reaction gave the fragment ions at *m*/*z* 315.08 and 289.07. These molecules are condensed products of (*epi*)catechin with acetaldehyde, and they correspond to two (*epi*)catechin moieties linked by an ethyl-bridge. Commonly, the biosynthesis of these compounds is due to electrophilic substitution by acetaldehyde on the nucleophilic C6 or C8 positions of catechins A-ring [28]. Compounds **21**, **28**, and **32** were tentatively identified as A-type procyanidins [29]. In particular, procyanidin A2, which was commonly found in unfermented red beans, was likely compound **28**, which showed the [M-H]^−^ ion at *m*/*z* 575.1198 and TOF-MS/MS fragment ions at *m*/*z* 557.1144 and 539.1008, which were attributable to the sequential loss of two water molecules. Compound **32** was suggested to be an isomer of the previous one, whereas the deprotonated molecular ion of compound **21** at *m*/*z* 591.1523 was proposed to be an (*epi*)catechin-(*epi*)gallocatechin [30]. A-type procyanidins were also compounds **18** and **24**, which were tentatively identified as 3T-*O*-β-D-galacto-pyranosyl-*ent*-(*epi*)catechin-(2α→7, 4α→8)-(*epi*)catechin, and 3T-*O*-L-arabinopyranosyl-*ent*-(*epi*)catechin-(2α→7, 4α→8)-(*epi*)catechin. These compounds were previously reported as a typical procyanidin compound in cocoa beans [31]. The TOF-MS/MS from (*epi*)catechin trimers **12**, **13**, and **20** at *m*/*z* 865.2 also showed the typical fragmentation pattern of B-type procyanidins, with the RDA fragment ions at *m*/*z* 713.15 and their dehydrated ions at *m*/*z* 695.14. The HRF reaction product consisted of the ions at *m*/*z* 739.17, whereas the (*epi*)catechin dimer at *m*/*z* 577.14 was derived from the QM reaction. Accordingly, the [M-H]^−^ ion for compound **19** was likely a B-type procyanidin tetramer. Compounds **16**, **25**, **26**, **27**, and **30** were flavonol glycosides, of which four shared quercetin as aglycone [32]. Compound **16** was identified by comparing the MS^2^ data and retention time with those of a pure reference compound. The loss of 132 Da suggested that compounds **25** and **27** were two isomers of quercetin pentoside differing in the sugar component identity, while compound **30** was likely quercetin rhamnoside. Finally, compound **26** exhibited kaempferol as aglycone, and rutinose disaccharide, based on the peculiar abundance of the aglycone radical ion, was localizable on the enolic function in the C-3 position. The content of flavonols is almost two-fold higher in ICB_hA_ than PCB_hA_.

#### 3.6.2. UHPLC-ESI-QqTOF-MS/MS Analyses of Lipophilic Extracts

**Figure 3:**
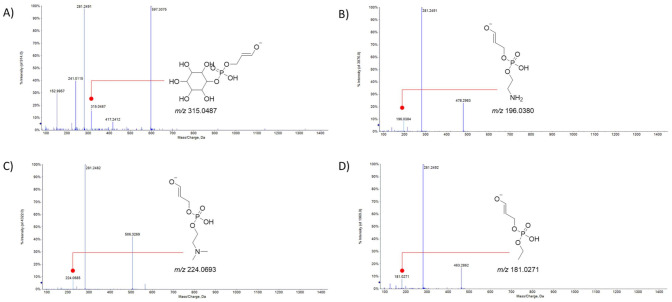
TOF-MS/MS spectra of compounds. TOF-MS/MS spectra of compounds **3′** (**A**), **5′** (**B**), **10′** (**C**), and **11′** (**D**). In the gray-colored boxes, diagnostic ions for the tentatively identified oleoyl-based lysophospholipids are reported. Theoretical *m*/*z* values are reported below each structure.

Organic fractions from biphasic extraction mainly consisted of fatty acids and their relative lyso-phospholipids. HR-MS/MS analyses confirmed that palmitic acid and stearic acid were the most representative saturated fatty acids, whereas oleic acid was the most abundant unsaturated fatty acid, followed by linoleic acid. All these fatty acyl moieties were found to be part of lysoglycerophospholipids, differing in the alcohol or amino-alcohol, which esterifies the phosphate group at the *sn3* position. In particular, UHPLC-ESI-Q*q*TOF-MS/MS analyses evidenced that the palmitoyl residue was in three different lysoglycerophospholipids. In fact, the [M-H]^−^ ion at *m*/*z* 571.2900 for compound **1′** (calcd. 571.2889 for C_25_H_49_O_12_P, error 1.9 ppm) was in accordance with a hexadecanoyl glycero-3-phosphoinositol. In TOF-MS/MS, the deprotonated molecular ion underwent inositol neutral loss to achieve the ion at *m*/*z* 391.2252, whereas following the loss of 316.0577 Da, the ion at *m*/*z* 255.2323, corresponding to dehydrated palmitic acid, was formed. The deprotonated molecular ion at *m*/*z* 452.2781 (C_21_H_43_NO_7_P) for compound **4′** equally gave rise to the ion at *m*/*z* 255.0386 (base peak), while the ion at *m*/*z* 196.0386 suggested that compound **4′** was a hexadecanoyl glycerol 3-phosphoethanolamine. Finally, palmitoyl moiety (at *m*/*z* 255.2315 as base peak) was also detected in the MS/MS spectrum of compound **9′**, whose deprotonated molecular ion was at *m*/*z* 480.3112. The detection of the ion at *m*/*z* 224.0662 allowed us to hypothesize that in this case, *N,N*-dimethylethanolamine is the amino alcohol esterifying the phosphate group. Compound **14′** was tentatively palmitic acid. Stearic acid, which was putatively identified in compound **16′**, represented the tail of lysophospholipids **7′** and **13′**. In particular, based on the neutral loss of 180.0694 Da (due to inositol) and the occurrence of the fragment ion at *m*/*z* 315.0486, compound **7′** with [M-H]^−^ ion at *m*/*z* 599.3226 was identified as an octadecanoyl glycero-3-phosphoinositol, while compound **13′** was characterized by the presence of *N,N*-dimethylethanolamine as an amino alcohol moiety. Lysophospholipids with a monounsaturated long-chain fatty acyl moiety such as oleic acid (18:1FA) were also detected (Figure 3). The [M-H]^−^ ion of the compound **3′** at *m*/*z* 597.3048 (calcd. 597.3045, according to the molecular formula C_27_H_51_O_12_P) provided, following the neutral loss of inositol the ions at *m*/*z* 417.2412, 315.0487 (C_9_H_16_O_10_P^−^), and 281.2491 (oleoate). This latter ion, together with the ion at *m*/*z* 196.0384, was also obtained as a base peak from the deprotonated molecular ion at *m*/*z* 478.2945 (compound **5′**). The ion at *m*/*z* 196.0384 was in line with the phosphatidylethanolamine derivative. Based on the fragment ion at *m*/*z* 224.0685, the compound **10′** showing the [M-H]^−^ ion at *m*/*z* 506.3246 was tentatively identified as an oleoyl glycerol 3-phospho-*N,N*-dimethylethanolamine. Furthermore, an oleoyl glycerol 3-phosphoethanol was putatively compound **11′**, which showed the deprotonated molecular ion at *m*/*z* 463.2837. The TOF-MS/MS experiment highlighted the fragment ions at *m*/*z* 281.2492 (base peak) and 181.0271. Compound **8′** was a hydroxy derivative of oleic acid. In fact, the deprotonated molecular ion at 299.2599, in accordance with the molecular formula C_18_H_36_O_3_, lost water to achieve the ion at *m*/*z* 281.2463. Compound **15′** was identified as oleic acid, based on comparison of its retention time and MS/MS data with those of the pure reference compound. Compounds **2′** and **6′** were likely linoleoyl glycerol 3-phosphoethanolamine and linoleoyl glycerol 3-phospho-*N,N*-dimethylethanolamine, respectively, whereas compound **12′** was linoleic acid (18:2, n-6).

## 4. Discussion

In recent years, the increasing interest in plant-derived healthy natural products has been focused on the research for normally consumed food and drinks whose natural chemical composition provides multi-purpose beneficial effects [11,33,34,35]. Thus, assuming that cocoa and derivatives are consumed frequently and widely all over the world as reported by Tokede and collaborators [36] due to the highly attractive organoleptic characteristics [4], herein the combined approach between the evaluation of various biological activities (radical scavenging, antimutagenicity, antigenotoxicity, and cancer cells viability inhibitory effect), and the chemical composition analysis by UHPLC-ESI Q*q*TOF-MS/MS of the selected cocoa beans was used. This allowed us to shed light on chemical and biological differences and/or similarities between marketed samples of cocoa beans var. Criollo coming from the geographical areas of Indonesia (ICB) and Peru (PCB). The cocoa bean is one of the most important agricultural export products of Indonesia [37], and it is one of the main farming activities in Peru. In particular, in Amazonas, in northern Peru, dry fermented Criollo cocoa beans, designed as Cacao Amazonas Peru, is produced for the Italian market [38]. The chemical composition of cocoa beans, as well as of all plant material in general, appeared to be affected by environmental conditions, which mainly influence the ratio of saturated to unsaturated fatty acids [39]. In line with findings by Torres-Moreno et al. [40], who, evaluating the fatty acids profile in cocoa and chocolates from Ecuador and Ghana, showed that the fatty acids profile varied depending on the different geographical origin, palmitic and stearic acids were the main saturated acids in cocoa beans herein investigated, where oleic acid (18:1) and linoleic acid (18:2) were the main unsaturated acids. The content of these four compounds slightly varied between the two samples. Indeed, while the content of stearic acid is comparable in ICB_O_ and PCB_O_ (16.2% and 16.9%, respectively), palmitic acid was 1.4-fold higher in ICB_O_ extract than in PCB_O_. This latter contained an appreciable amount of unsaturated fatty acids with oleic acid and oleate-containing lysophospholipids that represent 38.1% of the fatty acids in PCB_O_ extract. Without a doubt, the detection of the fatty acids in their unesterified form or as lysophospholipids is also attributable to tissues and cells damage occurring during extraction. The lysophospholipids content in cocoa beans was not valued anywhere in the literature; these common structural components of cell membranes are bioactive compounds that are able to affect carcinogenesis, neurogenesis, immunity, vascular development, or regulate metabolic diseases [41]. For instance, oleoyl-lysophosphatidylinositol was found to be an anti-diabetic substance that is able to induce the release of glucagon-like peptide-1 from L-cells with high potency by activating GPR-119 [42]. It is certain that the organic fraction from both the investigated beans lacks antiradical power, whereas a good scavenging capability was attributable to ICB_hA_ and PCB_hA_. In detail, the hydroalcoholic fraction of ICB showed a significantly better ability to scavenge both DPPH and ABTS radicals than that of PCB. The difference between ICB_hA_ and PCB_hA_ becomes more evident especially in scavenging the ABTS radical with a median effect concentration in the order of dozens and hundreds of μg/mL respectively when testing ICB_hA_ and PCB_hA_. The ability of the ABTS assay to detect the antiradical capacity of phytoderivatives better than the DPPH assay is also reported by Floegel and colleagues [43]. As observable from Table 5, ICB_hA_ compared to PCB_hA_ is characterized by the presence in higher percentage quantity especially of catechin (8, 22.07%) as the most present molecule, followed by isocitric acid (3, 8.94%), (*epi*)catechin ethyl dimer (31, 7.36%), caffeoyl aspartic acid (4, 6.98%) as well as all other *N*-phenylpropenoyl-L-amino acid–hydroxycinnamoyl amino acid conjugates (7, 10, 14, 22), procyanidin dimer A type (28, 5.71%), and although in a small percentage, by all (*epi*)catechin trimers (12, 13, 20). As reported by Grzesik and colleagues [44], catechins (catechin, epicatechin, epicatechin gallate, epigallocatechin, epigallocatechin gallate) are molecules with well-known antioxidant properties against ABTS and ROO free radicals, ferric ions, hypochlorite. The same authors underline that catechin (8, here found especially in ICB_hA_) was able to induce protection against the oxidation of dihydrorhodamina 123, protection against fluorescein bleaching by NaOCl or AAPH, and the inhibitory effect on AAPH-induced reactive oxygen species production in erythrocytes at concentrations in the order of units or tenths of μmol/L (tenths μg/mL) and lower than those obtained to determine the same activities using epicatechin (9, here found especially in PCB_hA_), explaining the higher ICB_hA_ antioxidant power obtained in this study. Furthermore, Grzesikand colleagues [44] underlined the best antioxidant activities of catechins also when compared to hydroxycinnamic acids and derivatives such as caffeic acid, chlorogenic acid, and hydrocinnamic acid. Apart from catechins, in 2001, Cartron and coauthors [45] underlined that caffeoyl derivatives showed specific antioxidant activity protecting low-density lipoproteins from oxidation and decreasing the pro-inflammatory lysophosphatidylcholine production. In the present study, different hydroxycinnamoyl amino acid conjugates, namely *N*-phenylpropenoyl-L-amino acids, were found, and among them, clovamide (10) was found in ICB_hA_ at 2.07%, which is 93.4% more than PCB_hA_. Zeng and collaborators [46] reported that clovamide exhibited anti-inflammatory properties, Tsunoda et al. [47] showed the anti-aggregant activity of clovamide toward amyloid β-protein implicated in Alzheimer’s disease and Fallarini et al. [48] antioxidant and neuroprotective effects of the same molecule. Certainly, considering the bioactivity data, particular attention must be given to the richness and diversity of hydroalcoholic cocoa beans fractions in A- and B-type procyanidins, which are however catechin oligomers. Liu and colleagues [49] demonstrated the strong scavenging effect on ·OH, and the IC_50_ values in the order of units of μg/mL, testing A-type dimeric and trimeric procyanidins, explaining that the antioxidant activity was related to the number of hydroxyls in their molecular structures. According to Kim and colleagues [50], procyanidins exert a wide range of beneficial effects including antioxidant activity, protection against DNA damage, and antitumor effects. In our study, when ICB (reached in procyanidin A type (28)) and PCB (reached in procyanidin B type like B_1_ (11) and B_4_ (15)) samples were co-incubated with standard mutagens, they showed significant moderate and strong antimutagenic effects, and similarly when they were co-incubated with standard genotoxins, they showed moderate antigenotoxic activity. Our results agree with those of López-Romero et al. [51] who showed that catechin, epicatechin, epigallocatechin, and epicatechin play a key role in antigenotoxic evidences. Furthermore, Yamagishi et al. [52], using cacao liquor extract, found an anticlastogenic effect against the formation of micronuclei induced by mitomycin C in mice bone marrow and peripheral blood cells; in 2016, Cajurao and Revale [53] observed an inhibitory potential of cacao against the genotoxicity induced by tetracycline in Swiss mice. As the cumulative DNA damage causes mutations involved also in the development of cancer, the correlation among free radical damage, genotoxic damage, and the potential development of cancer is not negligible [54]. The polyphenolic cocoa ingredients such as catechin, epicatechin, and procyanidin oligomers have a strong antioxidative activity and have a potential protective effect against cancer [55]. In this study, ICB_hA_ and PCB_hA_ inhibited the viability in the cancer cell lines selected at concentrations in the order of hundreds of μg/mL with the highest inhibition of cell viability observed for Hep-G2 and Caco-2. Comparing ICB_hA_ and PCB_hA_, ICB_hA_ showed the highest activity especially when tested on MCF-7 and Hep-G2. Our results are supported by the literature. Ramljak and coauthors [56] proved that procyanidins, in addition to determining G0/G1 cell cycle arrest, induced an inhibition of proliferation in human breast cancer cells at 100 μg/mL. In fact, procyanidins had already proved to exert cytotoxicity toward breast, colon, and prostate cancer cells at concentrations in the order of magnitude from units to hundreds of μg/mL [57,58,59]. In 2011, Ramos and colleagues [60] observed that epicatechin–gallate exerted anticarcinogenic effects on colon cancer SW480, leading them to apoptosis by caspase-3 activity, imbalance among Bcl-2 anto- and pro-apoptotic protein levels, ERK activation and AKT inhibition at concentrations higher than 20 μM (8.8 μg/mL). In this study, we observed that two Criollo var. of cocoa beans had differences in biological properties and chemical composition. In particular, the ICB_hA_ sample showed the highest radical scavenging, antimutagenicity, antigenotoxicity, and cancer cells viability inhibitory effects. Going back to the geographical origins of the two beans, Indonesia is the third biggest cocoa producer (777,750 MT/year) after Cote d’Ivoire and Ghana, with about a 15% share of total world cocoa bean production. Cocoa has been cultivated in Indonesia over 1.5 million hectares, generating over $1.2 billion in exports annually. Thus, the cocoa bean is one of the most important agricultural export products of Indonesia. Cocoa production provides the main source of income for over 1,400,000 smallholder farmers and their families in Indonesia, and they contribute 93% of national production [61]. In this area, precipitation is the dominant driver in cocoa success, so that high temperatures in humid tropical areas are ideal growing conditions for cocoa. On the other hand, Witjaksono [61] states that cocoa seedlings are particularly vulnerable to drought; hence, the water deficit can lead to low yields with a suffering production especially when there is decreased water availability due to elevated evapotranspiration or drought conditions coincide with higher temperatures. These just-described conditions along with many other climatic variations were observed in Peru since 2009, and they were reported by Keller and Echeverria [62] from the Peruvian International Institute for Sustainable Development. Nevertheless, although PCB was not overall the best sample, it however determined important proven biological properties. In the light of the above, as suggested by Kelloff et al. [63] and Martin et al. [4], the use of dietary components introduced through food, such as flavonoids (e.g., catechins and procyanidins), could help prevent, delay, and reduce the risk of cancer as well as of other chronic diseases. According to Arts et al. [64], the chocolate consumption contributed 2–5 mg of daily catechin intake. In 2006, Tabernero and collaborators [65] estimated for the Spanish diet that cocoa products account for 10% of the total antioxidant capacity of dietary intake. In addition, research conducted by Di Renzo and co-authors [66] reported the beneficial effects of the daily intake of 30 g of chocolate (70% cocoa) in pregnant women that showed reduced arterial pressure (diastolic and systolic). Moreover, the same authors highlighted a decrease in the risk of preclampsia, which is a major complication of pregnancy with cardiovascular manifestation that is related to chocolate consumption. Furthermore, as reported by Urbańska and colleagues [67], the systematic consumption of a piece of dark chocolate (10 g) is beneficial to the cardiovascular system and is therefore recommended as a component of everyday diet. In fact, already in a previous study performed by Sudarma and colleagues [68], the dark chocolate intake was useful against chronic degenerative diseases by reducing systolic pressure in prehypertension individuals and increasing nitric oxide levels as well as vasodilation, thus determining proven beneficial effects.

## 5. Conclusions

Investigating the metabolic profile of Criollo cocoa beans with different geographical origin, it was highlighted that they differed quantitatively in their polyphenol content. This latter mainly consists of proanthocyanids, flavonols, and *N*-phenylpropenoyl amino acids, whose diversity promptly strengths the dietary consumption of Criollo cocoa beans as a source of health-promoting compounds that are able to exert antioxidant, antimutagen, antigenotoxic, and anticancer properties. The chemical composition study is the starting point for further analyses, and it is aimed at deepening the availability of each Criollo bean as a source of bioactive compounds in the form of partially purified extracts, which are usable for supplementation purposes. Thus, based on the data herein acquired, fractionation strategies will be employed to better exploit Criollo bean polyphenol heritage and to deeper correlate the biological properties to the chemical composition, also considering interactions among individual molecules.

Furthermore, also in functional food perspectives, as the differences of the beans from the two geographical areas are not negligible, more research should be carried out to explore the impact of climatic variations (i.e., precipitation, temperatures, humidity) on growing conditions of cocoa. In fact, the comprehension of abiotic factors affecting cocoa polyphenols could favor their recovery and use in nutraceuticals and the food sector, also supporting policy initiatives aimed at increasing agricultural sustainable productivity and protecting biodiversity.

## Figures and Tables

**Table 1:** 2,2-Diphenyl-1-picrylhydrazyl (DPPH) and 2′-azino-bis (3-ethylbenzothiazoline-6-sulfonic acid) diammonium salt (ABTS) assays, concentration of samples to scavenge the ABTS^+^ of 50% (EC_50_) and Trolox Equivalent Antioxidant Capacity (TEAC) values. EC_50_ values (μg/mL) with confidence limits (95%, in brackets) and TEAC values (EC_50_ Trolox/EC_50_ Sample) of hydroalcoholic extracts of Indonesian cocoa beans (ICB) and Peruvian cocoa beans (PCB) samples in DPPH and ABTS assays. Trolox was used as standard.

	EC_50_		TEAC	
	DPPH	ABTS	DPPH	ABTS
**Trolox**	74.97 (64.31–87.40)	14.23 (12.40–16.30)	-	-
**ICB_hA_**	186.00 (165.00–209.80)	72.63 (67.66–77.84)	0.40	0.20
**PCB_Ha_**	289.30 (258.60–323.80)	322.20 (257.60–403.10)	0.26	0.04

**Table 2:** Antimutagenic activity. Antimutagenic activity of hydroalcoholic and chloroform extracts of ICB and PCB samples (10, 50, 100 μg/mL) in TA98 and TA100 strains after co-incubation with standard mutagens, respectively 2-nitrofluoren (2-NF, 2.5 and 10 μg/mL) and sodium azide (SOD, 5 and 20 μg/mL). Results (coming from three independent experiments) are expressed as mean of revertants/plates ± SD and as mean of inhibition rates % ± SD. Significant difference for ** *p * < 0.01 and *** *p* < 0.001 (*Dunnett’s test*) was calculated comparing extracts co-treated with standard mutagens to single standard mutagens.

Treatment [μg/mL]		TA98 Revertants/Plate (Mean ± SD)		Inhibition Rate (% Mean ± SD)		TA100 Revertants/Plate (Mean ± SD)		Inhibition Rate (% Mean ± SD)	
		2-NF ^3^ 2.5 μg/mL	2-NF 10 μg/mL	2-NF 2.5 μg/mL	2-NF 10 μg/mL	SOD ^4^ 5 μg/mL	SOD 20 μg/mL	SOD 5 μg/mL	SOD 20 μg/mL
**NC ^1^**		69 ± 7		-	-	232 ± 0		-	-
**M ^2^**		157 ± 8	646 ± 1	-	-	471 ± 9	1075 ± 7	-	-
**ICB_hA_**	**10**	39 ± 7 ***	391 ± 78 **	75 ± 3 ^b^	39 ± 12 ^a^	119 ± 10 ***	594 ± 37 ***	75 ± 2 ^b^	45 ± 3 ^b^
	**50**	27 ± 13 ***	352 ± 83 **	83 ± 7 ^b^	45 ± 13 ^b^	183 ± 13 ***	558 ± 36 ***	61 ± 3 ^b^	48 ± 3 ^b^
	**100**	17 ± 4 ***	217 ± 8 ***	89 ± 2 ^b^	66 ± 1 ^b^	142 ± 31 ***	530 ± 17 ***	70 ± 7 ^b^	51 ± 1 ^b^
**ICB_O_**	**10**	40 ± 0 ***	267 ± 34 ***	75 ± 1 ^b^	59 ± 5 ^b^	149 ± 1 ***	636 ± 66 ***	68 ± 0 ^b^	41 ± 6 ^b^
	**50**	31 ± 6 ***	246 ± 22 ***	80 ± 3 ^b^	62 ± 3 ^b^	114 ± 0 ***	621 ± 72 ***	76 ± 0 ^b^	42 ± 6 ^b^
	**100**	22 ± 4 ***	172 ± 15 ***	86 ± 2 ^b^	73 ± 2 ^b^	170 ± 28 ***	543 ± 18 ***	64 ± 7 ^b^	49 ± 1 ^b^
**PCB_hA_**	**10**	35 ± 3 ***	418 ± 82 **	78 ± 1 ^b^	35 ± 13 ^a^	143 ± 35 ***	630 ± 85 ***	70 ± 7 ^b^	41 ± 8 ^b^
	**50**	29 ± 6 ***	330 ± 79 ***	82 ± 3 ^b^	49 ± 12 ^b^	150 ± 57 ***	610 ± 70 ***	68 ± 11 ^b^	43 ± 6 ^b^
	**100**	20 ± 3 ***	169 ± 24 ***	87 ± 1 ^b^	74 ± 4 ^b^	188 ± 11 ***	544 ± 26 ***	60 ± 3 ^b^	49 ± 2 ^b^
**PCB_O_**	**10**	42 ± 11 ***	417 ± 38 **	73 ± 6 ^b^	35 ± 6 ^a^	141 ± 10 ***	638 ± 52 ***	70 ± 2 ^b^	41 ± 4 ^b^
	**50**	30 ± 8 ***	367 ± 87 **	81 ± 4 ^b^	43 ± 13 ^b^	112 ± 1 ***	586 ± 8 ***	76 ± 0 ^b^	46 ± 1 ^b^
	**100**	18 ± 6 ***	171 ± 16 ***	89 ± 3 ^b^	74 ± 2 ^b^	175 ± 18 ***	547 ± 35 ***	63 ± 5 ^b^	49 ± 4 ^b^

^1^ Negative control; ^2^ mutagen; ^3^ 2-nitrofluoren; ^4^ sodium azide; ^a^ moderate effect (25–40% inhibition), ^b^ Strong antimutagenic effect (>40% inhibition) [17].

**Table 3:** Antigenotoxicity of hydroalcoholic and chloroform extracts of ICB and PCB samples (25, 50, 100 μg/mL) after co-incubation with standard genotoxin 4-NQO (0.05 μg/mL). Results are expressed as mean of induction ratio (IR) ± SD (*n* = 3) with significant difference for * *p* < 0.05 and ** *p* < 0.01 (*Dunnett’s test*) calculated comparing IR values obtained from extracts co-treated with standard genotoxin to IR values obtained from single standard genotoxin, and as mean of antigenotoxicity % ± SD(*n* = 3).

	[μg/mL]	IR ± DS	Antigenotoxicity (% Mean ± SD)
**4-NQO ^1^**	**0.05**	2.31 ± 0.40	-
**ICB_hA_**	**25**	1.76 ± 0.39	24.15 ± 3.54 ^a^
	**50**	1.55 ± 0.21	32.40 ± 2.70 ^a^
	**100**	1.01 ± 0.16 **	56.26 ± 1.01 ^b^
**ICB_O_**	**25**	2.03 ± 0.32	26.05 ± 3.20 ^a^
	**50**	1.59 ± 0.28	36.11 ± 2.29 ^a^
	**100**	1.23 ± 0.18 *	58.30 ± 2.69 ^b^
**PCB_hA_**	**25**	1.72 ± 0.37	11.98 ± 1.35 ^a^
	**50**	1.48 ± 0.31	31.32 ± 0.01 ^a^
	**100**	0.96 ± 0.11 **	46.68 ± 1.78 ^b^
**PCB_O_**	**25**	1.93 ± 0.45	16.80 ± 5.30 ^a^
	**50**	1.59 ± 0.28	31.07 ± 0.24 ^a^
	**100**	1.18 ± 0.24 *	48.85 ± 1.42 ^b^

^1^ 4-Nitroquinoline. ^a^ neutral effect (<40% Antigenotoxicity). ^b^ moderate effect (40–70% antigenotoxicity). ^c^ strong effect (>70% antigenotoxicity) [20].

**Table 4:** 3-(4,5-Dimethylthiazol-2-yl)-2,5diphenyl-tetrazolium bromide (MTT) assay, concentration inhibiting the 50% cell growth rate (IC_50_) values. IC_50_ values (μg/mL) with confidence intervals of 95% (in brackets) obtained on MCF-7, OE19, Hep-G2, and Caco-2 human cell lines exposed to hydroalcoholic and chloroform extracts of ICB and PCB samples.

Cell Lines	IC_50_			
	ICB_hA_	ICB_O_	PCB_hA_	PCB_O_
**MCF-7**	254.20 (225.80–286.20)	>1000	708.30 (532.60–942.00)	>1000
**OE19**	903.30 (606.00–1347.00)	>1000	>1000	>1000
**Hep-G2**	122.00 (99.91–149.00)	>1000	199.70 (156.10–255.00)	>1000
**Caco-2**	104.90 (73.06–150.60)	181.50 (122.40–269.10)	133.90 (88.92–201.70)	234.00 (189.80–288.50)

**Table 5:** Time-of-flight mass spectrometry (TOF-MS) and TOF-MS/MS. TOF-MS and TOF-MS/MS of tentatively identified metabolites. RT = Retention Time; RDB = Ring Double Bond equivalent value.

	RT (min)	Tentative Assignment	Formula	[M-H]^−^ calc. (m/z)	[M-H]^−^ Found (m/z)	Error (ppm)	RDB	MS/MS Fragment Ions (m/z) and Relative Intensity (%)	ICB_hA_ Content (%)	PCB_hA_ Content (%)
**1**	0.275	Hexytol	C_6_H_14_O_6_	181.0718	181.0716	−0.9	0	113.0281, 101.0248 (100)	0.97	0.19
**2**	0.295	Disaccharide	C_12_H_22_O_11_	341.1094	341.1094	1.3	2	341.1094; 236.9674; 198.1265; 179.0553; 161.0459; 119.0363; 113.0252; 101.0253; 89.0254 (100)	1.4	1.66
**3**	0.360	Isocitric acid	C_6_H_8_O_7_	191.0197	191.0198	0.4	3	111.0087 (100); 87.0091	8.94	4.47
**4**	0.435 0.486	Caffeoyl aspartic acid	C_13_H_13_NO_7_	294.0619	294.0618 [2M-H]^−^ 589.1321	−0.4	8	206.0828; 204.0697; 178.0527; 161.0236; 135.0466; 134.0384; 132.0318 (100); 115.0048; 88.0416	6.98	4.62
**5**	0.636	Gallo(epi)catechin	C_15_H_14_O_7_	305.0667	305.0671	1.4	9	305.0671; 219.0667; 167.0353; 139.0403; 137.0248; 125.0248; 109.0299	1.42	1.65
**6**	0.615	Procyanidin B type (e.g., B_3_)	C_30_H_26_O_12_	577.1352	577.1361	1.6	18	577.1377; 559.1285; 451.1040; 425.0886; 407.0777 (100); 339.0856; 299.0572; 289.0705 (98); 287.0556; 245.0825; 221.0810; 167.0377; 161.0240; 125.0141	2.02	1.03
**7**	0.749	Coumaroyl aspartic acid	C_13_H_13_NO_6_	278.0670	278.0671	0.3	8	278.0672; 216.0653; 190.0857; 163.0382; 162.0560; 146.0610; 119.0503 (100); 117.0356; 115.0032; 93.0367	4.35	2.93
**8**	1.260	Catechin	C_15_H_14_O_6_	289.0718	289.0720 [2M-H]^−^ 579.1510	0.8	9	289.0718 (100); 245.0821; 203.0726; 179.0357; 125.0247; 109.0300	22.07	3.5
**9**	1.784	Epicatechin	C_15_H_14_O_6_	289.0718	289.0714	0.8	9	289.0723; 245.0825; 221.0833; 203.0730; 187.0414; 175.0418; 125.0252; 123.0461; 109.0308 (100); 97.0305	4.67	32.75
**10**	4.217	Clovamide	C_18_H_17_NO_7_	358.0932	358.0929	−0.9	11	358.0899; 222.0396; 178.0503; 161.0237; 135.0448 (100); 134.6163; 133.0301; 86.1043	2.07	1.07
**11**	0.800	Procyanidin B type (e.g., B_1_)	C_30_H_26_O_12_	577.1352	577.1363	2.0	18	577.1415; 559.1338; 451.1076; 425.0915; 407.0812 (100); 339.0900; 299.0568; 289.0738; 287.0578; 245.0837; 221.0829; 167.0361; 161.0255; 125.0254	2.08	10.25
**12**	4.255	((Epi)catechin trimer	C_45_H_38_O_18_	865.1985	865.1977	−0.9	27	739.1742; 713.1592; 695.1486; 577.1418; 559.1302; 543.0984; 525.0872; 451.1069; 425.0919; 413.0909; 407.0808; 381.1008; 341.0693; 289.0740; 287.0581; 261.0427; 243.0311; 217.0522; 175.0417; 161.0259; 125.0260	2.24	1.5
**13**	5.190	(Epi)catechin trimer	C_45_H_38_O_18_	865.1985	865.1977	−0.9	27	739.1739; 713.1583; 695.1451; 587.1247; 577.1409; 561.1076; 543.0971; 525.0885; 449.0892; 425.0908; 407.0787; 381.1008; 341.0687; 289.0728; 287.0575; 261.0417; 245.0463; 217.0530; 175.0402; 161.0256; 125.0249	4.46	3.86
**14**	5.661	Caffeoyl tyrosine	C_18_H_17_NO_6_	342.0983	342.0988	1.4	11	342.2398; 298.1083; 256.2008; 206.0473; 180.0675; 161.0242; 135.0452 (100); 119.0509; 107.0499; 93.0360	1.29	0.65
**15**	6.208	Procyanidin B type (e.g., B_4_)	C_30_H_26_O_12_	577.1352	577.1367	1.6	18	577.1374; 451.1046; 425.0892; 407.0774; 339.0871; 299.0554; 289.0716 (100); 287.0559; 245.0818; 161.0244; 125.02416	4.64	7.04
**16**	6.247	Isoquercetrin	C_21_H_20_O_12_	463.0882	463.0887	1.1	12	463.0884; 301.0340; 300.0267 (100); 271.0243; 255.0293; 243.0289	1.74	0.67
**18**	6.386	Procyanidin dimer A type hexoside isomer	C_36_H_34_O_17_	737.1737	737.1737	0.1	25	737.1780; 611.1448; 539.1018; 449.0899 (100); 448.0808; 407.0783; 388.0595; 327.0509; 307.0605; 287.0557	2.51	2.26
**19**	6.398	(Epi)catechin tetramer	C_60_H_50_O_24_	1153.2619	1153.2624	0.4	36	1153.2621; 1135.2792; 1001.2021; 983.2019; 575.1135; 289.0671; 287.0491; 245.0639; 161.0194; 125.0202	0.77	0.88
**20**	6.444	(Epi)catechin trimer	C_45_H_38_O_18_	865.1985	865.1977	−0.9	27	739.1762; 713.1582; 695.1488; 577.1423; 561.1118; 543.0994; 525.0885; 451.1085; 425.0915; 407.0814 (100); 381.1004; 299.0584; 289.0740; 287.0573; 245.0466; 161.0258; 125.0255	1.29	1.2
**21**	6.583	Proanthocyanidin A type [(epi)catechin-(epi)gallocatechin]	C_31_H_28_O_12_	591.1508	591.1523	2.5	18	591.2831; 591.1525; 591.2002; 547.1555; 439.1032; 301.0703; 289.0698 (100); 245.0814; 215.0702; 203.0698; 149.0226; 137.0236; 109.0280	1.22	1.6
**22**	6.623	p-Coumaroyl tyrosine	C_18_H_17_NO_5_	326.1034	326.1035	0.3	11	326.1046; 282.1143; 239.1091; 206.0461; 180.0664; 163.0401; 145.0292; 119.0498 (100); 117.0352; 93.0345	1.73	0.89
**23**	6.655	(Epi)catechin ethyl dimer	C_32_H_30_O_12_	605.1665	605.1685	3.4	18	605.1675; 453.1197; 315.0875 (100); 289.0719; 271.0961; 245.0819; 229.0875; 205.0503; 163.0404; 151.0396; 137.0244; 109.0300	1.71	2.58
**24**	6.670	Procyanidin dimer A type pentoside isomer	C_35_H_32_O_16_	707.1618	707.1636	2.6	20	707.1663; 581.1345; 539.1020; 449.0895 (100); 448.0815; 407.0789; 325.0339; 287.0543; 125.0246	2.39	1.98
**25**	6.682	Quercetin pentoside	C_20_H_18_O_11_	433.0776	433.0776	12	−0.1	433.0776; 301.0344; 300.0274 (100); 271.0248; 255.0302; 227.0354; 199.0407; 178.9991; 151.0024; 107.0138		
**26**	6.702	Kaempferol rutinoside	C_27_H_30_O_15_	593.1512	593.1523	13	1.9	593.1512 (100); 593.2491; 549.2540; 447.0976; 429.0806; 285.0396; 284.0314	0.35	0.43
**27**	6.783	Quercetin pentoside	C_20_H_18_O_11_	433.0776	433.0775	12	−0.1	433.0775; 301.0345; 300.0272 (100); 271.0239; 255.0288; 243.0288; 227.0339; 151.0025	0.23	0.04
**28**	6.959	Procyanidin dimer A type	C_30_H_24_O_12_	575.1195	575.1198	19	0.5	575.1236; 557.1144; 539.1008; 531.2619; 471.1195; 449.0897; 423.0719; 409.0994; 407.0784; 387.0591; 341.0620; 327.0501; 307.0631; 289.0729; 287.0547; 285.0414 (100); 267.0306; 241.0501; 217.0504; 163.0044; 161.0252; 137.0259; 125.0246; 109.0315	5.71	3.74
**29**	7.041	Procyanidin B type (e.g., B_2_)	C_30_H_26_O_12_	577.1352	577.1360	1.5	18	577.1369; 425.0891; 407.0878; 289.0720 (100); 287.0565; 245.0819; 161.0239; 125.0244	0.55	0.67
**30**	7.144	Quercetin deoxyhexoside (e.g., Q-rhamnoside)	C_21_H_20_O_11_	447.0933	447.0945	12	2.7	447.0959; 301.0361; 300.0285 (100) 283.0246; 271.0251; 255.0304; 243.0301; 227.0349; 211.0406; 178.9981; 151.0036; 121.0289	0.4	0.55
**31**	8.280	(Epi)catechin ethyl dimer	C_32_H_30_O_12_	605.1665	605.1673	1.4	18	605.1693; 453.1168; 315.0867; 289.0714 (100); 271.0956; 245.0811; 229.0856; 205.0504; 179.0347; 163.0416; 151.0397; 137.0250; 109.0293	7.36	2.98
**32**	8.422	Proanthocyanidin A type	C_30_H_24_O_12_	575.1195	575.1207	19	2.1	575.1215 (100); 449.0887; 407.0778; 394.0692; 287.0565; 271.0239; 243.0287; 229.0504; 161.0230; 137.0245; 125.0245	1.02	0.71

## Supplementary Materials

The following are available online at https://www.mdpi.com/2304-8158/10/3/571/s1, Figure S1: Concentration/effect curves, Table S1: Mutagenicity and genotoxicity.
