# Supplementary material for: Theobromacacao Criollo var. Beans: Biological Properties and Chemical Profile
Source: Foods. 2021 Mar 9;10(3):571. doi: 10.3390/foods10030571 (PMC8001065; doi:10.3390/foods10030571)
Supplement: Supplementary file 1 [file foods-10-00571-s001.pdf]

Theobroma cacao Criollo var. beans from two geographical areas: antiradical, antiproliferative, antigenotoxic and antimutagenic activities of their chemically profiled extracts

Margherita Lavorgna, Roberta Nugnes, Chiara Russo*, Elena Orlo, Simona Piccolella, Severina Pacifico**, Marina Isidori

Dipartimento di Scienze e Tecnologie Ambientali, Biologiche e Farmaceutiche, Università degli Studi della Campania "Luigi Vanvitelli", Via Vivaldi 43, I-81100 Caserta, Italy

*****Corresponding author:

Chiara Russo: chiara.russo@unicampania.it

******Corresponding author:

Severina Pacifico: severina.pacifico@unicampania.it

Figure S1. Concentration/effect curves obtained relating inhibition of cell viability % to concentration [μg/mL] of hydroalcoholic extracts of ICB and PCB samples on MCF-7, OE19, Hep-G2 and Caco-2 human cell lines.

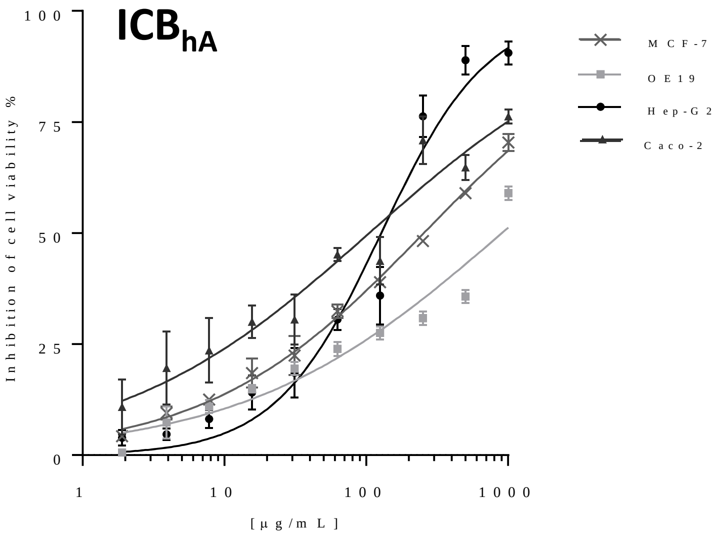

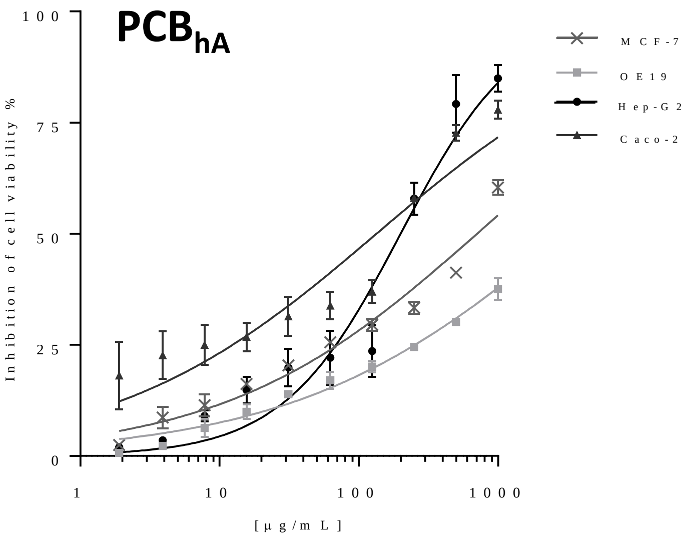

ICB Indonesian cocoa beans; **PCB** Peruvian cocoa beans.

Table S1. Mutagenicity and genotoxicity of hydroalcoholic and chloroform extracts of ICB and PCB samples (1000 μg/mL) in TA98, TA100 strains and in TA1535/pSK1002 strain, respectively. Results are presented as mutagenic ratio (MR) and induction ratio (IR) and are expressed as mean ± standard deviation (SD) of three independent experiments. No mutagenic effect (MR<2); No genotoxic effect (IR<1.5).

**Treatment [μg/mL]**	**RM (mean ± SD)**		**IR (mean ± SD)**
	**TA98**	**TA100**	**TA1535/pSK1002**
**2-NF 2.5**	2.30 ± 0.12	-	
**2-NF 5**	5.03 ± 0.10	-	
**2-NF 10**	9.45 ± 0.95	-	
**SOD 5**	-	2.03 ± 0.04	
**SOD 10**	-	3.02 ± 0.01	
**SOD 20**	-	4.64 ± 0.03	
**4-NQO**	-	-	2.14 ± 0 .07
**ICB_{hA}**	1.41 ± 0.20	1.84 ± 0.11	0.65 ± 0.04
**ICB_{O}**	1.50 ± 0.36	1.91 ± 0.02	0.64 ± 0.35
**PCB_{hA}**	1.76 ± 0.07	1.80 ± 0.06	0.53 ± 0.06
**PCB_{O}**	1.30 ± 0.19	1.92 ± 0.10	0.72 ± 0.24
